# Flock-level risk factors for scrapie in Great Britain: analysis of a 2002 anonymous postal survey

**DOI:** 10.1186/1746-6148-2-25

**Published:** 2006-08-03

**Authors:** K Marie McIntyre, Simon Gubbins, S Kumar Sivam, Matthew Baylis

**Affiliations:** 1 Institute for Animal Health, Pirbright Laboratory, Ash Road, Pirbright, Surrey GU24 0NF, UK; 2 Veterinary Laboratories Agency, New Haw, Addlestone, Surrey KT15 3NB, UK; 3 Veterinary Clinical Science, University of Liverpool, Leahurst, Neston, Wirral, Cheshire CH64 7TE, UK

## Abstract

**Background:**

In November 2002, an anonymous postal survey of sheep farmers in Great Britain (GB) was conducted to identify factors associated with the flock-level occurrence of scrapie. This survey was undertaken to update an earlier postal survey in 1998, and was the first occasion in which a large-scale postal survey had been repeated.

**Results:**

The results of the 2002 survey indicated that scrapie was more likely to occur in certain geographic regions; in purebred compared to commercial flocks; in larger flocks; in flocks which lambed in group pens compared to those which lambed in individual pens; in flocks which always lambed in the same location compared to those which did not; and in farms which kept certain breeds of sheep. In addition to these factors, the likelihood of the disease occurring in homebred animals was higher in flocks which bred a greater proportion of replacement animals or which bought-in lambs. Finally, within-flock transmission following exposure was more likely to occur in hill flocks compared to other farm types; in flocks which bred a greater proportion of replacement animals; and in farms which kept a certain crossbreed of ewe.

**Conclusion:**

The risk factors identified from the 1998 and 2002 anonymous postal surveys in Great Britain were similar. However, differences between the surveys were identified in the influence of region and of purchasing behaviour on the risk of scrapie. These differences are most likely a consequence of changes in farmer awareness and the impact of the 2001 foot-and-mouth disease epidemic, respectively.

## Background

In 1997 the Spongiform Encephalopathy Advisory Committee (SEAC) recommended that a postal survey of sheep farmers in Great Britain (GB), to which farmers could respond anonymously, should be carried out [1]. The first such survey was undertaken in 1998 [2-4], and aimed to improve estimates of the incidence of scrapie and overcome the perceived unwillingness of some farmers to report the disease.

Farmer-based surveys have been used to estimate the prevalence and incidence of scrapie in GB [2,3,5], The Netherlands [6], Ireland [7] and the Shetland Isles [8]. Where surveys are used to collect information on sheep demography and farm management, flock-level risk factors for scrapie occurrence can also be investigated [3,4,8]. In particular, the 1998 survey identified several risk factors [3,4]. Flock size, geographical region, lambing practices and keeping certain sheep breeds were all shown to influence the risk of scrapie occurring in a flock. Further analysis indicated that the likelihood of scrapie occurring in homebred animals was influenced by the proportion of replacement animals bred on a farm. Finally, scrapie was more likely to be transmitted within a flock, following exposure, in farms which bought-in large numbers of rams, purchased few ewes and bred a high proportion of replacement animals.

To update the findings of the 1998 survey a further postal survey was undertaken in November 2002 [9]. This was the first occasion in which a large-scale postal survey had been repeated. The design of the 2002 survey was based on that run in 1998, but also took into account the impact of the 2001 foot-and-mouth disease epidemic in GB, the introduction of the National Scrapie Plan for GB (NSP) in 2001 and asked more refined questions in certain areas of interest. The 2002 survey also coincided with the introduction of large-scale surveillance for scrapie, including testing of animals found-dead on farm [10] and animals slaughtered for human consumption [11].

Estimates for the prevalence and incidence of scrapie based on the 2002 survey have been reported elsewhere [9,12] and here we focus on identifying flock-level risk factors for disease. Importantly, the 2002 survey gathered information that allowed us to examine risk factors for both the acquisition of infection and its subsequent spread within a flock. For our purposes, farms that acquire infection have their first case in a purchased animal; farms with subsequent transmission have cases in homebred animals.

## Results

### (i) Risk factors for scrapie to occur in a flock (never versus ever)

Comparing farms that have ever had scrapie (n = 331) with those which have never had the disease (n = 2180) ('never versus ever' in Table 2) indicated that all regions in England had similar odds of having scrapie; the risk of having disease was 3.4 times higher in Shetland (SH); and it was lower in central and southern Scotland (SC(CS)) and in Wales (W(N), W(CS)). Commercial flocks had a lower risk of scrapie than purebred ones. Flocks in which ewes were lambed in individual pens had a lower risk of disease than those in which ewes were lambed in group pens in a building, while flocks which always lambed in the same location had a higher risk than those which sometimes lambed in the same location. Keeping crossbred Charollais ewes, Suffolk rams or Swaledale rams was associated with an increased risk of having scrapie, while keeping Blackface rams was associated with a decreased risk. An interaction between flock size and stocking density was found, which indicated that the risk of scrapie increased significantly with flock size only when the stocking density was relatively high (third quartile).

### (ii) Risk factors for homebred cases to occur in a flock (never versus homebred)

For farms that have had scrapie in at least one homebred animal (n = 96) there was little regional variation in risk, except in Shetland (SH) where the odds of having a homebred case were 7.5 times higher ('never versus homebred' in Table 2). Commercial farms were less likely to have homebred cases than purebred farms. The risk of homebred scrapie increased 1.4-fold for each 10% increase in the proportion of home-bred animals. The risk was also higher if lambs were bought-in to the farm; or if ewes were lambed in group pens in a building. Keeping Suffolk ewes, Swaledale ewes or Charollais rams was associated with an increased risk of homebred scrapie while keeping Bluefaced Leicester ewes or Beulah Speckled Face rams were associated with a decreased risk.

An interaction between flock size and stocking density was identified and included in the final model. This implied that the risk of scrapie in homebred animals increased significantly with flock size only when the stocking density was relatively high (third quartile). A further interaction was identified between flock size and lambing practices. However, the frequencies for some combinations of factor levels were zero or very small (with correspondingly large standard errors for the coefficients) and, therefore, this interaction was not included in the final model.

### (iii) Risk factors for transmission to occur within a flock (purchased versus homebred)

Comparing farms that have had scrapie in homebred animals (n = 96) with those which have had cases in purchased animals only (n = 204) indicated that the risk of transmission occurring within a flock increased 1.4-fold for each 10% increase in the proportion of homebred animals ('purchased versus homebred' in Table 2). Transmission was less likely to occur in lowland compared with hill flocks, while keeping crossbred Texel ewes was associated with a decreased risk of a within-flock outbreak.

For each of the models a Hosmer-Lemeshow goodness-of-fit test [13] indicated an acceptable fit of the model to the data (Table 2).

## Discussion

This paper provides an update of the results of an earlier postal survey conducted in 1998 [3,4], but has further explored regional effects in Scotland and Wales; the effect of the temporary housing on and then return of sheep from other farms; the confounding effects of the location and its regularity of use when lambing on the farm; and whether or not popular ram breeds or crossbreeds were kept on farms.

The robustness of the survey depends on the ability of farmers to recognise scrapie in their animals. In an attempt to minimise farmer misdiagnosis, a leaflet on the clinical signs of disease was included with the questionnaire and, of those farmers who reported having scrapie in their flock, only 2.9% gave inappropriate answers to questions on clinical signs of scrapie [3,12].

There was significant regional variation in the risk of scrapie (Table 2), as was also observed in the 1998 survey [4]. In 2002, the risk of ever having scrapie was slightly higher in south east England (SE) and the West Midlands (WM), and lower in Yorkshire and Humberside (YH) than in 1998. In the analysis of both surveys scrapie occurrence is not limited to any time period and, hence, the changes in the estimates are unlikely to reflect temporal changes in scrapie risk, rather they may reflect changes in farmer awareness of scrapie.

Flock size has often been identified as an important risk factor for scrapie, with an increase in flock size associated with an increase in risk [3,4,8,14,15]. An effect of flock size was also identified in this study (Table 2), but only in an interaction with stocking density. At higher stocking densities (second quartile or above) the risk of scrapie increases with flock size as expected, though the increase is statistically significant only for flocks in the third quartile. By contrast, there appears to be no association with flock size for flocks in the first quartile; an effect that is accounted for by an unexpectedly high number of small flocks stocked at low density which reported scrapie. This could reflect a bias in the data, by which farmers with small flocks are able to observe their flocks more closely and, hence, are more likely to spot disease. An interaction between flock size and stocking density was noted in the 1998 survey [4], but the authors did not give sufficient detail to allow any comparison to be made.

Farm type reflects the land on which a flock grazes, while flock type reflects the primary business of the flock, namely the production of breeding stock (purebred) or lambs for human consumption (commercial). The effects of farm and flock type were similar in both the 1998 and 2002 surveys (Table 2; cf. [4]). There was a lower risk of having scrapie in commercial flocks compared with purebred flocks and a higher risk of within-flock transmission following exposure in hill flocks compared with other farm types. The difference in risk between commercial and purebred flocks reflects differences in flock management. Sheep spend a smaller amount of time in commercial flocks before being sold on and, consequently, it is less likely that disease would be observed. Differences in the risk of within-flock transmission in hill compared with other farm types may be a result of management practices specific to hill flocks. Alternatively, it could reflect a higher frequency of scrapie susceptible prion protein (PrP) genotypes in hill flocks, primarily related to sheep breeds kept on these farms [16].

The movement of animals has often been identified as an important mechanism for the transmission of scrapie between flocks [3,4,14,15,17]. The results of the 2002 survey suggest that buying-in lambs carries a greater risk than buying-in rams or ewes. This agrees with the results of the Shetland survey [8], but differs from the 1998 survey where buying-in rams increased, and buying-in ewes decreased the odds of scrapie [4]. The differences between the results for the 1998 and 2002 surveys most likely reflect the impact of the 2001 foot-and-mouth disease epidemic in GB on purchasing behaviour in the twelve months preceding the 2002 survey. Compared with the 1998 survey, fewer farms reported purchasing rams or ewes while more reported purchasing lambs in the 2002 survey. This identifies a potential shortcoming of the survey in that the data on purchasing behaviour are based on the previous twelve months, while scrapie occurrence is not limited to a particular time period, thus making comparison between surveys difficult.

Flock management related to lambing influenced the risk of scrapie. In particular, an increase in the proportion of replacement animals bred on the farm was associated with a greater risk of homebred cases within the flock, as was also observed in the 1998 survey [4]. In flocks where sheep were lambed in individual pens in a building, the risk of scrapie was lower compared with those in which sheep were lambed in group pens in a building. Furthermore, flocks which always lambed in the same location had a higher risk of scrapie than flocks which did not. The association between lambing practices and scrapie risk reflects the probable role in transmission of infectivity in the placenta [18,19] and foetal membranes [20,21]. Consequently, scrapie transmission is likely to be affected by the duration and proximity of contact between sheep at lambing.

Whether or not certain breeds and crossbreeds of ewes and rams were kept on a farm influenced the risk of scrapie (Table 2); something also observed in the 1998 survey [4]. Two breeds (Blackface and Swaledale) were identified as having the same impact on scrapie risk in both surveys, but the remaining breeds differed between the surveys. The impact of breed on scrapie risk may reflect the influence of breed-specific management practices, but could also arise because of intrinsic differences in risk amongst sheep breeds. These intrinsic differences could reflect differences in the PrP genotype profile amongst breeds (see [16] for example) or some other breed effect, possibly not associated with the PrP gene. However, it is important to stress that the postal survey is based on the past, farmer-diagnosed, occurrence of scrapie over an unspecified time-period. The reported breed differences reflect, therefore, historical differences and take little account of changes in the susceptibility of breeds as a result of recent breeding programmes and, in particular, the NSP. They categorically do not imply that the presence of these breeds increases the risk of scrapie occurring now.

The relationship between breed, PrP genotype and scrapie risk raises a potential shortcoming of this study, namely that it ignores the effect of the PrP genotype profile of a flock on the acquisition and within-flock transmission of scrapie. Although PrP genotype strongly influences the risk of individual animals developing clinical disease [22,23], the impact of PrP genotype on risk at a flock-level remains to be determined (see [24] for example). Modelling studies, however, suggest that the frequency of PrP genotypes will be a significant factor in a within-flock outbreak of scrapie [25,26]. A further risk factor not explored is the influence of feeding practices, which a recent study has suggested may affect the occurrence of scrapie [27].

## Conclusion

Similar flock-level risk factors for scrapie were identified within the 1998 and 2002 anonymous postal surveys in Great Britain. Important risk factors include geographical region, flock type (purebred or commercial), flock size, sheep breed, trading practices and the use of certain lambing practices.

The results of the two surveys differed in the influences of specific geographical regions and purchasing of lambs on the risk of scrapie. Rather than reflecting changes in scrapie risk, these differences are most likely a consequence of changes in farmer awareness and the impact of the 2001 foot-and-mouth disease epidemic, respectively.

## Methods

Full details of the survey are presented elsewhere [12] and here we present only a summary of the design. In November 2002 questionnaires were sent to a random sample of sheep farmers registered on the 2001 Agricultural Census as keeping at least 30 breeding ewes. The sample was stratified by country (England, Scotland and Wales) based on the number of flocks. Of 12800 questionnaires sent out, 6791 were returned and entered on the database. Farms entered on the database were excluded from the final analyses if they did not keep sheep; if they did not know or did not state whether or not scrapie had occurred in the flock; or if they did not provide information for all potential risk factors included in the analyses. Possible biases that could result from excluding these farms were explored, but none were identified. 

We first explored the results using univariate analyses to identify potential risk factors and decide appropriate coding for the variables. The list of attributes and their interpretation is given in Table 1. After this, multivariate techniques were employed. Three main comparisons were made between farms. First, to identify the risk factors for scrapie to occur, farmers reporting ever having had a case were compared to those never reporting the disease (never versus ever). Second, farmers reporting cases in at least one homebred sheep were compared to those never having reported the disease, thus identifying risk factors for scrapie to occur in homebred animals (never versus homebred). Third, farmers reporting cases in at least one homebred animal were compared to those in which cases were reported only in purchased sheep, to identify risk factors for transmission to occur within a flock following exposure to the infectious agent (purchased versus homebred). A further comparison of farmers who reported never having scrapie with those who reported the disease in purchased animals only (never versus purchased) was considered, but the results did not lead to any further insight and so have not been presented.

Binary logistic regression models were used to explore relationships between scrapie occurrence and a farm's attributes. Models were built using forward stepwise regression through the first twelve attributes listed in Table 1, followed by the inclusion of all possible pairwise interactions. Once the minimal adequate model was reached, the most popular breeds and crossbreeds of rams and ewes (those reported on over 150 farms) were added to the model. Statistical significance was determined by a *P*-value of less than 0.05. Adjusted odds ratios (AOR) significantly different from one were used as an indicator of raised or lowered risk of scrapie. The goodness-of-fit of the final model was judged using a Hosmer-Lemeshow goodness-of-fit test [13].

## Authors' contributions

KMM carried out the statistical analyses and drafted the manuscript. SG carried out the statistical analyses and critically revised the manuscript. SKS and MB conceived and designed the study and critically revised the manuscript. All authors read and approved the final manuscript.

## Figures and Tables

**Figure 1:**
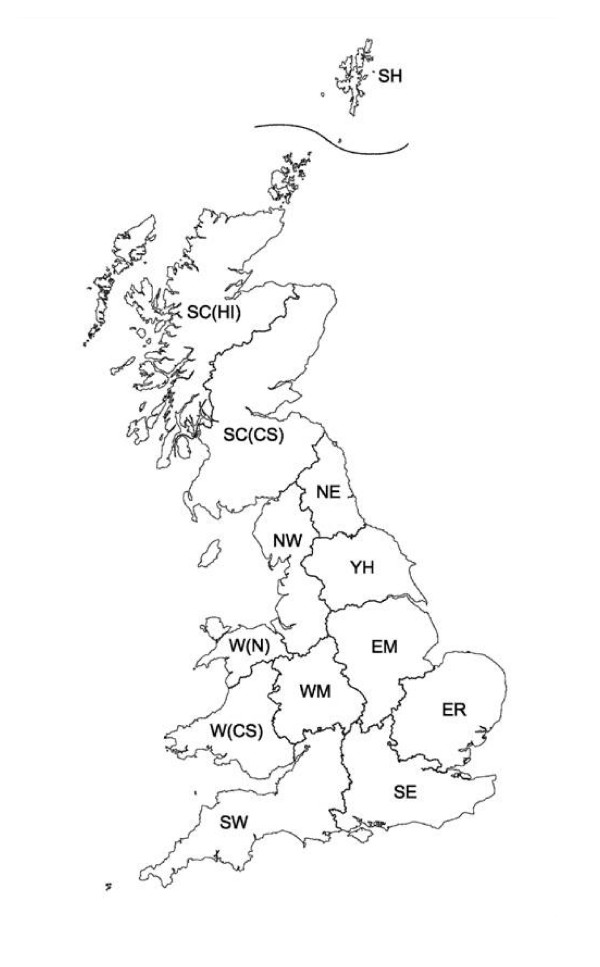
**Regions in GB used to code the attribute 'region' in Tables 1 & 2**. South-west England (SW), south-east England (SE), eastern England (ER), East Midlands (EM), West Midlands (WM), Yorkshire and Humberside (YH), north-west England (NW), north-east England (NE), central and south Scotland (SC(CS)), Scottish Highlands and Islands (SC(HI)), Shetland Isles (SH), North Wales (W(N)) and central and south Wales (W(CS)).

**Table 1:** Potential risk factors for scrapie considered in the analyses

Attribute	Description, coding and comments
region	Great Britain divided into the 13 regions indicated in figure 1
farm type	hill (H); upland (U); lowland (L)
flock type	purebred (P); commercial (C); both (PC)
flock size	log(number of rams and ewes over one year old)
stocking density	quartiles for stocking density (number of rams and ewes over one year old divided by the number of acres grazed): <0.85 (Q1); <1.56 (Q2); <2.51 (Q3); >2.51 (Q4)
proportion homebred	the proportion of rams and ewes over one year old born on the farm; the AOR gives the change in odds for an increase of 10% in the proportion of homebred animals
rams bought-in	did (1) or did not (0) purchase rams in the previous 12 months
ewes bought-in	did (1) or did not (0) purchase ewes in the previous 12 months
lambs bought-in	did (1) or did not (0) purchase lambs in the previous 12 months
sheep temporarily moved elsewhere	sheep did (1) or did not (0) temporarily spend time on other farms in the last 12 months (e.g. over-wintering, summer grazing, fattening)
lambing practices	sheep lambed: in individual pens in a building (1); unconfined at pasture (2); in group pens at pasture (3); in group pens in a building (4); did not lamb in the previous 12 months (5)
lambing location	sheep lambed: always in the same location (1); sometimes in the same location (2); never in the same location (3)
breeds kept on farm	most popular breeds and crossbreeds of ewes and rams (those reported on over 150 farms); coded as do (1) or do not (0) keep breed on the farm

**Table 2:** Logistic regression models for scrapie risk (Adjusted odds ratios (AOR) shown in bold differ significantly (*P *< 0.05) from one)

attribute	never versus ever			never versus homebred			purchased versus homebred		
	
	AOR	95% CL		AOR	95% CL		AOR	95% CL	
						
		lower	upper		lower	upper		lower	upper
*region*									
East Midlands	baseline	-	-	baseline	-	-	**-**	**-**	**-**
eastern England	0.90	0.34	2.37	1.32	0.12	14.42	**-**	**-**	**-**
north-east England	0.63	0.30	1.31	0.55	0.10	2.95	**-**	**-**	**-**
north-west England	1.05	0.55	2.00	1.17	0.26	5.31	**-**	**-**	**-**
central and south Scotland	**0.29**	0.14	0.60	0.18	0.03	1.07	**-**	**-**	**-**
Scottish Highlands & Islands	0.53	0.25	1.12	0.74	0.16	3.35	**-**	**-**	**-**
south-east England	1.18	0.59	2.37	0.71	0.12	4.15	**-**	**-**	**-**
Shetland Isles	**3.42**	1.55	7.57	**7.54**	1.66	34.20	**-**	**-**	**-**
south-west England	0.83	0.46	1.48	1.39	0.36	5.45	**-**	**-**	**-**
central and south Wales	**0.34**	0.18	0.63	0.79	0.20	3.14	**-**	**-**	**-**
North Wales	**0.25**	0.11	0.58	0.30	0.05	1.76	**-**	**-**	**-**
West Midlands	0.74	0.39	1.41	1.05	0.23	4.91	**-**	**-**	**-**
Yorkshire and Humberside	1.46	0.79	2.68	1.53	0.34	6.84	**-**	**-**	**-**
*farm type*									
hill	-	-	-	-	-	-	baseline	-	-
upland	-	-	-	-	-	-	0.42	0.18	1.01
lowland	-	-	-	-	-	-	**0.35**	0.18	0.66
*flock type*									
purebred	baseline	-	-	baseline	-	-	-	-	-
commercial	**0.55**	0.37	0.81	**0.46**	0.24	0.86	-	-	-
both	1.35	0.84	2.17	1.70	0.82	3.51	-	-	-
*flock size interacting with stocking density*									
first quartile	1.06	0.84	1.33	0.93	0.67	1.29	-	-	-
second quartile	1.69	0.96	2.98	1.96	0.80	4.81	-	-	-
third quartile	**2.13**	1.17	3.89	**3.75**	1.27	11.06	**-**	-	-
fourth quartile	1.58	0.90	2.75	1.96	0.80	4.77	-	-	-
*proportion homebred*	-	-	-	**1.36**	1.22	1.50	**1.37**	1.24	1.52
*lambs bought-in*									
no	**-**	**-**	**-**	baseline	-	-	-	-	-
yes	**-**	**-**	**-**	**2.13**	1.16	3.89	-	-	-
*lambing practices*									
individual pens in building	baseline	**-**	**-**	baseline	-	-	-	-	-
unconfined at pasture	1.37	0.80	2.35	3.46	0.94	12.78	-	-	-
group pens at pasture	1.89	0.81	4.42	3.70	0.63	21.66	-	-	-
group pens in building	**1.90**	1.17	3.10	**5.09**	1.44	17.92	-	-	-
did not lamb	2.33	0.88	6.21	**16.63**	2.45	113.0	-	-	-
*lambing location*									
always the same place	baseline	-	-	-	-	-	-	-	-
sometimes the same place	**0.51**	0.29	0.90	-	-	-	-	-	-
never the same place	1.83	0.48	7.00	-	-	-	-	-	-
*ewe breeds*									
Suffolk	-	-	-	**2.21**	1.32	3.68	-	-	-
Texel cross	-	-	-	-	-	-	**0.34**	0.14	0.86
Charollais cross	**2.98**	1.92	4.63	-	-	-	-	-	-
Bluefaced Leicester	**-**	-	-	**0.34**	0.13	0.93	**-**	-	-
Swaledale	**-**	-	-	**8.56**	3.40	21.60	-	-	-
*ram breeds*									
Suffolk	**1.58**	1.20	2.07	-	-	-	-	-	-
Blackface	**0.25**	0.09	0.75	**-**	-	-	-	-	-
Charollais	-	-	-	**2.55**	1.33	4.91	-	-	-
Beulah Speckled Face	-	-	-	0.20	0.04	1.00	-	-	-
Swaledale	**2.95**	1.58	5.50	-	-	-	-	-	-
Hosmer-Lemeshow goodness-of-fit test	*P *= 0.41			*P = *0.18			*P *= 0.94		
